# Systematic Review and Clinical Outcomes of new Robotic Systems in Urology

**DOI:** 10.1590/S1677-5538.IBJU.2025.0007

**Published:** 2025-03-25

**Authors:** Iulia Andras, Federico Piramide, Carlo Andrea Bravi, Fabrizio Di Maida, Filippo Turri, Edward Lambert, Mike Wenzel, Danny Darlington, Marco Paciotti, Giuseppe Basile, Christoph Wurnschimmel, Nikolaos Liakos, Gabriele Sorce, Ruben De Groote, Marcio Covas Moschovas, Paolo Dell’Oglio, Nicolae Crisan, Alexandre Mottrie, Alessandro Larcher

**Affiliations:** 1 Iuliu Hatieganu University of Medicine and Pharmacy Department of Urology Cluj-Napoca Romania Department of Urology, Iuliu Hatieganu University of Medicine and Pharmacy, Cluj-Napoca, Romania; 2 University of Turin San Luigi Gonzaga Hospital Department of Oncology Turin Orbassano Italy School of Medicine, Division of Urology, Department of Oncology, San Luigi Gonzaga Hospital, University of Turin, Orbassano, Turin, Italy;; 3 The Royal Marsden NHS Foundation Trust Department of Urology London UK Department of Urology, The Royal Marsden NHS Foundation Trust, London, UK;; 4 University of Florence Careggi Hospital Department of Urology Florence Italy Department of Urology, Careggi Hospital, University of Florence, Florence, Italy; 5 University of Milan Department of Urology, ASST Santi Paolo e Carlo Milan Italy Department of Urology, ASST Santi Paolo e Carlo, University of Milan, Milan, Italy;; 6 ORSI Academy OLV Hospital, Aalst, Belgium Department of Urology Belgium Department of Urology, OLV Hospital, Aalst, Belgium; ORSI Academy, Melle, Belgium;; 7 Goethe University Frankfurt University Hospital Frankfurt Department of Urology Frankfurt am Main Germany Department of Urology, University Hospital Frankfurt, Goethe University Frankfurt, Frankfurt am Main, Germany;; 8 Royal Surrey County Hospital Department of Urology, Stokes Centre for Urology Guildford UK Department of Urology, Stokes Centre for Urology, Royal Surrey County Hospital, Egerton Road, Guildford, GU2 7XX, UK;; 9 Vita-Salute San Raffaele University Department of Urology Milan Italy Department of Urology, Vita-Salute San Raffaele University, 08025 Milan, Italy;; 10 Lucerne Cantonal Hospital Department of Urology Lucerne Switzerland Department of Urology, Lucerne Cantonal Hospital, Lucerne, Switzerland;; 11 Medical Faculty and Medical Centre of the University of Freiburg Department of Urology Freiburg Germany Department of Urology, Medical Faculty and Medical Centre of the University of Freiburg, Freiburg, Germany;; 12 AdventHealth Global Robotics Institute Kissimmee FL USA AdventHealth Global Robotics Institute, Kissimmee, FL, USA;; 13 ASST Grande Ospedale Metropolitano Niguarda Department of Urology Milan Italy Department of Urology, ASST Grande Ospedale Metropolitano Niguarda, Milan, Italy;; 14 Department of Urology and Division of Experimental Oncology Milan Italy Department of Urology and Division of Experimental Oncology, URI, Urological Research Institute, IRCCS San Raffaele Scientific Institute, Milan, Italy.

**Keywords:** Robotic Surgical Procedures, Systematic Reviews as Topic, General Surgery

## Abstract

**Purpose:**

The adoption of novel multi-port, single-port and modular robotic platforms has significantly increased in the last years. We aim to provide an overview of the preliminary clinical outcomes of the procedures performed with these new robotic systems, assessing their particular features and safety profile during the learning curve

**Material and methods:**

A systematic literature search was performed on 15th May 2023 on PubMed, Embase, Scopus and Web of Science databases, to identify original articles presenting clinical outcomes of new robotic systems for abdominal urologic surgery. The study protocol was registered on PROSPERO (CRD 42023437863).

**Results:**

Six new robotic platforms were identified. Of 2925 papers identified, 71 met our inclusion criteria: 49 on single-port system and 22 on novel multi-port systems. We found variable outcomes for the most common procedures performed with these new systems. However, all of them showed acceptable perioperative and oncologic outcomes during the learning curve and good safety profile. Functional outcomes were underreported

**Conclusions:**

The adoption of novel multi-port and single-port robotic systems in urologic surgery can offer new opportunities for enhanced precision, reduced invasiveness, and potentially improved patient outcomes. The variability in outcomes across different platforms underscores the need for continued research and standardized training.

## INTRODUCTION

The adoption of novel multi-port, single-port and modular robotic platforms has significantly increased in the last years (1). The majority of these new systems are currently installed as additional platforms in centers where DaVinci multiport systems are already available. However, in a few settings they represent the first robotic system available, as an alternative to laparoscopy or the traditional open approach (2, 3).

The lower costs entailed by the competition between new robotic systems (4), together with a potentially improved surgical robustness have led to this wide implementation. The novel multiport systems bring forward several features that could enhance the operating room experience: the open console is potentially more ergonomic and facilitates communication between the surgeon and the other team members (5), the independent modular robotic arms allow for a more flexible positioning (6) and the possibility of haptic feedback overcomes some concerns regarding the standard DaVinci platform.

Moreover, the single-port system decreases even more the invasivess of the surgical procedures and allows the development of innovative types of approach, such as transvesical for radical prostatectomy (7) or supine-anterior retroperitoneal approach for upper urinary tract surgery (8), regionalizing the surgery and leading to the extension of the surgical indications in complex patients with previous abdominal procedures.

The urologic surgery landscape has already changed by the adoption of these new platforms. The aim of this systematic review is to show an overview of the preliminary clinical outcomes of the procedures performed with new robotic systems (multi-port and single-port), assessing their particular features and safety profile at the beginning of the learning curve of these new platforms. The importance of the current paper resides in the fact that it provides the first comprehensive analysis of similar procedures performed multi-port and single-port systems, aiming to act as an initial basis for decision-making in centers wanting to increase their number of robotic systems.

## MATERIALS AND METHODS

### Search strategy

A systematic literature search was performed on 15th May 2023 on PubMed, Embase, Scopus and Web of Science databases, to identify articles on new robotic systems. The study protocol was registered on PROSPERO (registry number CRD 42023437863).

The search strategy used the PICO criteria (9):

□ P (population): patients undergoing transperitoneal or retroperitoneal abdominal urologic surgery
□ I (intervention): transperitoneal/ retroperitoneal abdominal urologic surgery using robotic systems (multi-port and single-port) approved after 2014 (alternatives to the standard DaVinci Xi or SI)
□ C (comparator): transperitoneal/retroperitoneal urologic surgeries performed with different types of new robotic systems (multi-port and single-port) approved after 2014 (alternatives to the standard DaVinci Xi or SI)
□ O (outcome): perioperative, functional and oncologic outcomes
□ S (study design): original studies

The search string is available in Supplementary file 1.

### Article selection

**Figure 1:**
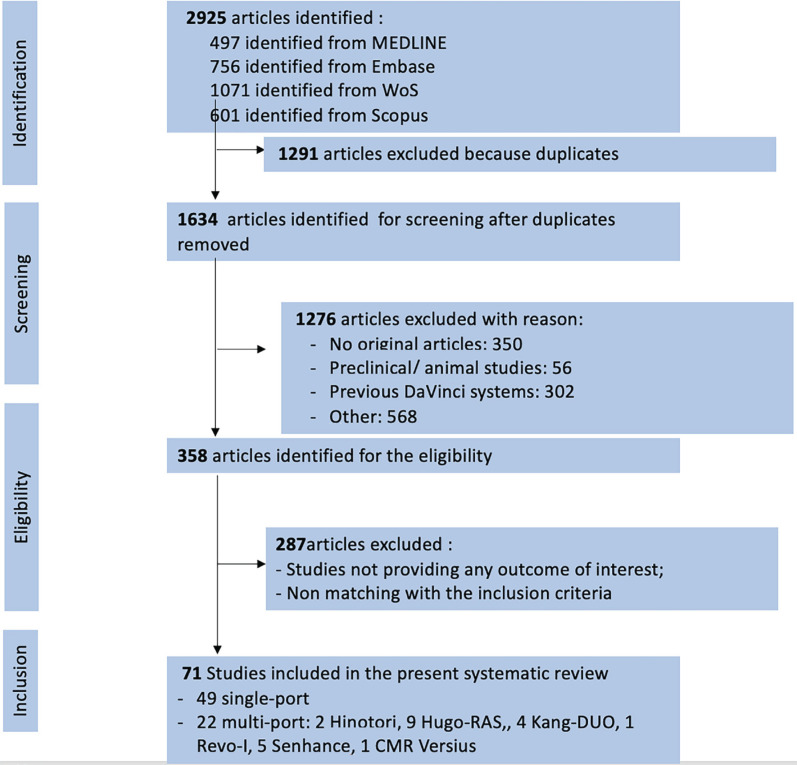
PRISMA flow diagram for study screening and selection.

Articles were selected according to the Preferred Reporting Items for Systematic Reviews and Meta-analyses (PRISMA) (10) (Figure-1). We included in the current review all English-language original papers presenting clinical evidence on new robotic platforms used for abdominal urologic surgery. Letter to the editor, review articles, preclinical studies, studies including animals or cadavers and studies not presenting outcomes of interest were excluded. Video articles and brief correspondence papers were included if they presented clinical outcomes of interest.

Four authors (CB, EL, FM, FT) independently screened the articles based on the inclusion and exclusion criteria. In case of disagreement, another author helped to reach consensus (MW). Similarly, the selected papers were assessed for eligibility by four authors (CB, DD, FM, MP) and another author helped to reach consensus (FP, IA). References from the selected articles were manually reviewed to identify supplementary relevant studies. To avoid duplication of patients, in case of series from the same center, the paper with the highest number of cases was considered.

### Risk-of-bias assessment

All papers included in the present systematic review were assessed for the risk of bias using the ROBINS-I tool for non-randomized studies of intervention by three authors (EL, NL, MW) (11).

### Assessment of study quality and level of evidence

Study quality was assessed by three authors (FT, CW, DD) based on the Newcastle-Ottawa scale (12) and the level of evidence was assessed according to the Oxford Centre for Evidence-Based Medicine (13).

### Data extraction and analysis

Data was extracted into an a *priori* created database by 4 authors (IA, FP, MW, GS). Continuous variables reported as median (range) were converted to the mean ± standard deviation (SD) using a designated formula (14). If the parametric distribution was unknown (i.e., the mean and SD couldn't be calculated), those data were excluded from the pooled analysis. After converting to mean ± SD, the data were further expressed as the mean with a 95% confidence interval (CI). The weighted mean was then used to calculate the overall mean for variables measured in multiple studies. Proportions were used to represent categorical variables. Data were graphically displayed when possible. No further comparative analyses were performed.

### Types of new robotic systems

Our search identified 6 multiport novel robotic system and one single-port system already used in the clinical practice. The characteristics of these systems are presented hereby.

### Hinotori

Hinotori system, developed by Medicaroid Corporation (Japan), consists of a console, patient and video cart which are similar to DaVinci system. However, Hinotori provides an additional degree of freedom for the arms and computer-control over their movement, aiming to minimize external conflicts (15). Also, the robotic arms are not docked with the ports, increasing the available space for the bedside surgeon (16).

### Hugo-RAS

Hugo-RAS, a robotic system developed by Medtronic, is the platform available most frequently after the standard Da Vinci system. This system has several novel features (17), such as the open console with 3D screen, which facilitates the communication between the surgeon and the team members. Moreover, it a modular system, consisting in 4 independent arms allowing for a more versatile configuration. The tilting and docking angles can be set differently for each arm, in order to avoid external collision and allow access to multiple anatomic quadrants. The hand-controllers have a „pistol-like" design, which can improve the surgical gesture ergonomy. Another important feature is the built-in computer and dedicated app able to record all surgical procedures and perform automated analytics.

### Kang-DUO

Kang-DUO is a self-developed robotic system by SuZhou Kang Duo Robot, China. It consists in an open console, a three-arm surgical cart and a video tower. The novelty of this system is represented by the passive polarizing 3D glasses, which enable the control of robotic instruments without flexion of the surgeon's neck (18). The 3D display system is compatible with various 3D laparoscopic camera producers, which can potentially reduce the costs (19).

### Revo-I

Revo-I is a robotic platform developed by Meerecompany, Korea. Its components are similar to the DaVinci multiport platform, with a closed console, patient cart with 4 robotic arms and video cart. Collision warning messages are integrated in this platform in order to overcome the lack of haptic feedback (20).

### Senhance

Senhance is another novel multiport modular robotic platform, developed by TransEnterix Inc. It has an open console configuration, with hand-controllers similar to the laparoscopic instruments. It uses standard non-wristed laparoscopic instruments with haptic sensing incorporated. The eye-tracking camera movement and head controlled zoom motion are important features of this system (21).

### Versius CMR

Versius platform is the second most commonly used new robotic multiport platform in Europe after Hugo-RAS. It is a modular system with four separate arms. The surgeon console is open with a 3D screen. The instrument tips have seven degrees of freedom. A novel feature included in this system is the transfer of all the controls onto the handheld joysticks, removing the need for foot pedal control (22).

### Da Vinci single-port

The Da Vinci single port system developed by Intuitive™ was available only in the US at the end of 2018, after FDA approval. Recently, it is also available in several high-volume centers in Europe. The aim of this system is to decrease the surgical morbidity and invasiveness, but at the same time increase the versatility and allow access in difficult surgical sites. It consists of a single trocar through which a flexible camera and three bi-articulated instruments are introduced (23). The surgeon console is similar to the previous Da Vinci systems.

## RESULTS

### Overview of the included studies

The initial search identified a total of 2925 papers. After screening and eligibility assessment, 71 studies met the inclusion criteria and were included in the current review: 49 on single-port system and 22 on multi-port systems. Six novel multi-port systems already used in clinical practice were identified: Hinotori, Hugo-RAS, Kang-DUO, Revo-I, Senhance and Versius. The majority of the included studies are retrospective case series, with only one randomized-controlled trial and few propensity-score matched analyses.

### Bias assessment, level of evidence and study quality

All the included studies were rated to have intermediate quality according to Newcastle-Ottawa scale. The quality assessment and level of evidence for the included studies is reported in Supplementary Table-1. Risk of bias assessment is presented in Supplementary Table-2.

**Supplementary Table 1:** Evaluation of study quality and level of evidence of the included studies.

Authors	Type of robotic system	Type of study (prospective/ retrospective)	Type of study	Study quality	Level of evidence
Miyake, et al. 2023 (24)	Hinotori	prospective	Case series	poor	4
Hinata, et al. 2022 (25)	Hinotori	retrospective	Case series	poor	4
Totaro, et al. 2022 (26)	Hugo	retrospective	Case series	poor	4
Alfano, et al. 2023 (27)	Hugo	retrospective	Case series	poor	4
Balestrazzi, et al. 2024 (28)	Hugo	retrospective	Comparative un-matched	poor	4
Bravi, et al. 2023 (29)	Hugo	retrospective	Case series	poor	4
Ellorieta, et al. 2023 (30)	Hugo	retrospective	Case series	poor	4
Gallioli, et al. 2023 (31)	Hugo	prospective	Case series	poor	4
Mottaran, et al. 2023 (32)	Hugo	retrospective	Case series	poor	4
Raffaeli, et al. 2023 (33)	Hugo	retrospective	Case series	poor	4
Ragavan, et al. 2023 (34)	Hugo	retrospective	Case series	fair	4
Fan, et al. 2021 (35)	Kang-DUO	prospective	Case series	poor	4
Fan, et al. 2022 (36)	Kang-DUO	prospective	Case series	poor	4
Dong, et al. 2023 (37)	Kang-DUO	prospective	Case series	poor	4
Li, et al. 2023 (38)	Kang-DUO	prospective	RCT	good	1
Alip, et al. 2022 (39)	Revo-I	prospective	Case series	fair	4
Kastelan, et al. 2021 (40)	Senhance	prospective	Case series	poor	4
Venckus, et al. 2021 (41)	Senhance	prospective	Case series	poor	4
Knezevic, et al. 2022 (42)	Senhance	retrospective	Case series	poor	4
Kulis, et al. 2022 (43)	Senhance	prospective	Case series	fair	4
Sassani, et al. 2022 (44)	Senhance	retrospective	Case series	fair	4
Hussein, et al. 2023 (45)	Versius	retrospective	Case series	good	4
Dobbs, et al. 2019 (46)	Da Vinci SP	prospective	Case series	poor	4
Heo, et al. 2019 (47)	Da Vinci SP	retrospective	Case series	poor	4
Ng, et al. 2019 (48)	Da Vinci SP	prospective	Case series	poor	4
Steinberg, et al. 2019 (49)	Da Vinci SP	retrospective	Case series	poor	4
Steinberg, et al. 2020 (50)	Da Vinci SP	retrospective	Case series	poor	4
Agarwal, et al. 2020 (51)	Da Vinci SP	retrospective	Case series	Fair	4
Na, et al. 2020 (52)	Da Vinci SP	retrospective	Case series	poor	4
Fang, et al. 2020 (53)	Da Vinci SP	retrospective	Case series	poor	4
Francavilla, et al. 2022 (54)	Da Vinci SP	retrospective	Case series	poor	4
Ganesan, et al. 2020 (55)	Da Vinci SP	retrospective	Case series	poor	4
Huang, et al. 2021 (56)	Da Vinci SP	retrospective	comparative	Poor	4
Jones, et al. 2020 (57)	Da Vinci SP	retrospective	Case series	poor	4
Kaouk et al 2020 (58)	Da Vinci SP	retrospective	comparative	Good	3
Kim, et al. 2020 (59)	Da Vinci SP	retrospective	Case series	Poor	4
Saidian, et al. 2020 (60)	Da Vinci SP	retrospective	comparative	Fair	3
Shukla, et al. 2021 (61)	Da Vinci SP	retrospective	Case series	poor	4
Vigneswaran, et al. 2020 (62)	Da Vinci SP	retrospective	Case series	fair	4
Wilson, et al. 2020 (63)	Da Vinci SP	retrospective	Case series	poor	4
Garden, et al. 2021 (64)	Da Vinci SP	retrospective	Case series	poor	4
Lee, et al. 2021 (65)	Da Vinci SP	retrospective	Matched-comparison	good	3
Lenfant, et al. 2021 (66)	Da Vinci SP	retrospective	Matched-comparison	poor	4
Abou Zeinab, et al. 2022 (67)	Da Vinci SP	retrospective	Case series	poor	4
Abou Zeinab, et al. 2022 (68)	Da Vinci SP	retrospective	Matched-pair analysis	good	3
Balasubramanian, et al. 2022 (69)	Da Vinci SP	retrospective	Comparative unmatched	poor	4
Bassett, et al. 2022 (70)	Da Vinci SP	retrospective	Case series	Poor	4
Beksac, et al. 2022 (71)	Da Vinci SP	retrospective	Case series	poor	4
Covas Moschovas, et al. 2022 (72)	Da Vinci SP	retrospective	Case series	poor	4
Fang, et al. 2023 (73)	Da Vinci SP	retrospective	Comoparative unmatched	poor	4
Fracavilla, et al. 2022 (74)	Da Vinci SP	retrospective	Case series	poor	4
Ganesan, et al. 2022 (75)	Da Vinci SP	retrospective	Propensity-matched	fair	4
Harrison, et al. 2022 (76)	Da Vinci SP	retrospective	Propensity-matched	good	3
Harrison, et al. 2022 (77)	Da Vinci SP	retrospective	Propensity-matched	good	3
Kaouk, et al. 2022 (78)	Da Vinci SP	retrospective	Case series	Poor	4
Kaviani, et al. 2022 (79)	Da Vinci SP	retrospective	Case series	poor	4
Kim, et al. 2022 (80)	Da Vinci SP	retrospective	Propensity-matched	Good	3
Kim, et al. 2022 (81)	Da Vinci SP	retrospective	Case series	Poor	4
Koukouris, et al. 2022 (82)	Da Vinci SP	Prospective	Case series	Poor	4
Lee, et al. 2022 (83)	Da Vinci SP	retrospective	Matched-comparison	Good	3
Levy, et al. 2023 (84)	Da Vinci SP	retrospective	Case series	Poor	4
Liu, et al. 2022 (85)	Da Vinci SP	retrospective	Case series	Poor	4
Noh, et al. 2022 (86)	Da Vinci SP	retrospective	Case series	poor	4
Palacios, et al. 2022 (87)	Da Vinci SP	retrospective	Case series	poor	4
Ramos-Carpinteyro, et al. 2023 (88)	Da Vinci SP	retrospective	Case series	poor	4
Bang, et al. 2023 (89)	Da Vinci SP	retrospective	Comparative unmatched	poor	4
Harrison, et al. 2023 (90)	Da Vinci SP	retrospective	Propensity-matched	good	3
Kaouk, et al. 2023 (91)	Da Vinci SP	retrospective	Case series	Poor	4
Kim, et al. 2023 (92)	Da Vinci SP	retrospective	Case series	fair	4
Oh, et al. 2023 (93)	Da Vinci SP	retrospective	Case series	poor	4
Tyson, et al. 2023 (94)	Da Vinci SP	retrospective	Case series	poor	4

**Supplementary Table 2:** Risk of bias analysis of the included studies.

Studies	Type of robotic system	Confounding	Selection bias	Classification of interventions bias	Deviations from intended interventions bias	Missing data	Measurement of outcomes bias	Selection of the reported result bias
Miyake, et al. 2023 (24)	Hinotori	High risk	Low risk	Low risk	Low risk	Low risk	Some concerns	Some concerns
Hinata, et al. 2022 (25)	Hinotori	High risk	High risk	Low risk	Low risk	Low risk	Some concerns	Some concerns
Totaro et al. 2022 (26)	Hugo	High risk	Low risk	Low risk	Low risk	High risk	Some concerns	Some concerns
Alfano, et al. 2023 (27)	Hugo	High risk	Low risk	Low risk	Low risk	High risk	Low risk	Some concerns
Balestrazzi, et al. 2024 (28)	Hugo	Some concerns	Some concerns	Low risk	Low risk	Low risk	Low risk	Low risk
Bravi, et al. 2023 (29)	Hugo	High risk	Low risk	Low risk	Low risk	Low risk	Low risk	Some concerns
Elorrieta, et al. 2023 (30)	Hugo	High risk	High risk	Low risk	Some concerns	High risk	Some concerns	High risk
Gallioli, et al. 2023 (31)	Hugo	High risk	Low risk	Low risk	Some concerns	Low risk	Low risk	Some concerns
Mottaran, et al. 2023 (32)	Hugo	High risk	Some concerns	Low risk	Low risk	Low risk	Low risk	Some concerns
Raffaelli, et al. 2023 (33)	Hugo	High risk	Some concerns	Low risk	Low risk	Low risk	Low risk	Some concerns
Ragavan, et al. 2023 (34)	Hugo	High risk	High risk	Low risk	Low risk	High risk	Some concerns	High risk
Fan, et al. 2021 (35)	Kang-DUO	High risk	High risk	Low risk	Low risk	Low risk	Some concerns	Some concerns
Fan, et al. 2022 (36)	Kang-DUO	High risk	High risk	Low risk	Low risk	Low risk	Low risk	Low risk
Dong, et al. 2023 (37)	Kang-DUO	High risk	High risk	Low risk	Low risk	Low risk	Some concerns	Some concerns
Li, et al. 2023 (38)	Kang-DUO	High risk	High risk	Low risk	Low risk	High risk	Some concerns	High risk
Alip, et al. 2022 (39)	Revo-I	Some concerns	High risk	Low risk	Low risk	High risk	Some concerns	Some concerns
Kastelan, et al. 2021 (40)	Senhance	High risk	High risk	Low risk	Low risk	Low risk	Some concerns	Some concerns
Venckus, et al. 2021 (41)	Senhance	High risk	Low risk	Low risk	Low risk	Low risk	Some concerns	Some concerns
Knezevic, et al. 2022 (42)	Senhance	High risk	High risk	Low risk	Low risk	High risk	Low risk	High risk
Kulis, et al. 2022 (43)	Senhance	High risk	Low risk	Low risk	Low risk	Low risk	Some concerns	Some concerns
Sassani, et al. 2022 (44)	Senhance	High risk	High risk	Low risk	Low risk	High risk	High risk	Some concerns
Hussein, et al. 2023 (45)	Versius	High risk	Some concerns	Low risk	Some concerns	High risk	Low risk	Some concerns
Dobbs, et al. 2019 (46)	Da Vinci SP	High risk	Low risk	Low risk	Low risk	Low risk	Low risk	Some concerns
Heo, et al. 2019 (47)	Da Vinci SP	High risk	High risk	Low risk	Low risk	Low risk	Low risk	Some concerns
Ng, et al. 2019 (48)	Da Vinci SP	Some concerns	High risk	Low risk	Low risk	Low risk	High risk	High risk
Steinberg, et al. 2019 (49)	Da Vinci SP	Some concerns	High risk	Low risk	Low risk	Some concerns	Some concerns	High risk
Steinberg, et al. 2020 (50)	Da Vinci SP	Some concerns	Low risk	Low risk	Low risk	Low risk	Low risk	Some concerns
Agarwal, et al. 2020 (51)	Da Vinci SP	High risk	Some concerns	Some concerns	Low risk	Low risk	High risk	High risk
Na, et al. 2020 (52)	Da Vinci SP	Some concerns	High risk	Some concerns	Some concerns	Low risk	Some concerns	Some concerns
Fang, et al. 2020 (53)	Da Vinci SP	High risk	High risk	Low risk	Low risk	High risk	Low risk	High risk
Francavilla, et al. 2022 (54)	Da Vinci SP	High risk	High risk	Low risk	Low risk	High risk	Some concerns	Some concerns
Ganesan, et al. 2020 (55)	Da Vinci SP	High risk	High risk	Low risk	Low risk	Some concerns	Some concerns	Some concerns
Huang, et al. 2021 (56)	Da Vinci SP	Some concerns	Some concerns	Low risk	Low risk	Low risk	High risk	High risk
Jones, et al. 2020 (57)	Da Vinci SP	Some concerns	Some concerns	Low risk	Low risk	Some concerns	Some concerns	High risk
Kaouk, et al. 2020 (58)	Da Vinci SP	Some concerns	Some concerns	Low risk	Low risk	Low risk	Some concerns	High risk
Kim, et al. 2020 (59)	Da Vinci SP	Some concerns	High risk	Low risk	Low risk	Low risk	High risk	High risk
Saidian, et al. 2020 (60)	Da Vinci SP	Some concerns	Some concerns	Low risk	Low risk	Low risk	Some concerns	Some concerns
Shukla, et al. 2021 (61)	Da Vinci SP	Some concerns	Some concerns	Low risk	Low risk	Some concerns	Low risk	Low risk
Vigneswaran, et al. 2020 (62)	Da Vinci SP	Some concerns	High risk	Some concerns	Low risk	Low risk	Some concerns	Some concerns
Wilson, et al. 2020 (63)	Da Vinci SP	Some concerns	High risk	Low risk	Low risk	Low risk	Some concerns	Some concerns
Garden, et al. 2021 (64)	Da Vinci SP	Low risk	Some concerns	Low risk	Low risk	Low risk	Low risk	Low risk
Lee, et al. 2021 (65)	Da Vinci SP	Some concerns	High risk	Some concerns	Low risk	Low risk	Low risk	Some concerns
Lenfant, et al. 2021 (66)	Da Vinci SP	Some concerns	Some concerns	Low risk	Low risk	Low risk	High risk	High risk
Abou Zeinab, et al. 2022 (67)	Da Vinci SP	Some concerns	Some concerns	Low risk	Low risk	Low risk	High risk	High risk
Abou Zeinab, et al. 2022 (68)	Da Vinci SP	Low risk	Some concerns	Low risk	Low risk	Low risk	Low risk	Low risk
Balasubramanian, et al. 2022 (69)	Da Vinci SP	Low risk	Some concerns	Low risk	Low risk	Low risk	Some concerns	Some concerns
Bassett, et al. 2022 (70)	Da Vinci SP	Some concerns	Some concerns	Low risk	Low risk	Low risk	Some concerns	High risk
Beksac, et al. 2022 (71)	Da Vinci SP	High risk	High risk	Low risk	Low risk	Low risk	High risk	High risk
Covas Moschovas M, et al. 2022 (72)	Da Vinci SP	High risk	High risk	Low risk	Low risk	High risk	Some concerns	Some concerns
Fang AM, et al. 2023 (73)	Da Vinci SP	High risk	High risk	Low risk	Low risk	High risk	Some concerns	High risk
Francavilla, et al. 2022 (74)	Da Vinci SP	High risk	Some concerns	Some concerns	Low risk	Low risk	Low risk	Low risk
Ganesan, et al. 2022 (75)	Da Vinci SP	Some concerns	Low risk	Low risk	Low risk	Some concerns	Low risk	Some concerns
Harrison, et al. 2022 (76)	Da Vinci SP	Low risk	Some concerns	Some concerns	Low risk	Low risk	Some concerns	Some concerns
Harrison, et al. 2022 (77)	Da Vinci SP	Low risk	Low risk	Low risk	Low risk	Some concerns	Low risk	Low risk
Kaouk, et al. 2022 (78)	Da Vinci SP	High risk	High risk	Low risk	Low risk	Low risk	Some concerns	Some concerns
Kaviani, et al. 2022 (79)	Da Vinci SP	Some concerns	Some concerns	Low risk	Low risk	Some concerns	Some concerns	High risk
Kim, et al. 2022 (80)	Da Vinci SP	Low risk	Some concerns	Low risk	Low risk	Low risk	Low risk	Some concerns
Kim, et al. 2022 (81)	Da Vinci SP	Some concerns	High risk	Some concerns	Low risk	Low risk	Some concerns	Some concerns
Koukouris, et al. 2022 (82)	Da Vinci SP	Some concerns	High risk	Low risk	Low risk	Low risk	High risk	High risk
Lee, et al. 2022 (83)	Da Vinci SP	High risk	High risk	Some concerns	Low risk	Low risk	Some concerns	High risk
Levy, et al. 2023 (84)	Da Vinci SP	Some concerns	High risk	Some concerns	Low risk	Low risk	High risk	High risk
Liu, et al. 2022 (85)	Da Vinci SP	Some concerns	High risk	Low risk	Low risk	Low risk	High risk	High risk
Noh, et al. 2022 (86)	Da Vinci SP	Some concerns	High risk	Some concerns	Low risk	Low risk	Some concerns	High risk
Palacios, et al. 2022 (87)	Da Vinci SP	High risk	High risk	Some concerns	Low risk	Low risk	Low risk	Low risk
Ramos-Carpinteyro, et al. 2023 (88)	Da Vinci SP	Some concerns	Some concerns	Low risk	Low risk	Low risk	Low risk	Low risk
Bang, et al. 2023 (89)	Da Vinci SP	Some concerns	High risk	Low risk	Low risk	Low risk	Some concerns	Some concerns
Harrison, et al. 2023 (90)	Da Vinci SP	Low risk	Some concerns	Low risk	Low risk	Low risk	Some concerns	High risk
Kaouk, et al. 2023 (91)	Da Vinci SP	Low risk	Some concerns	Low risk	Low risk	Low risk	Some concerns	Some concerns
Kim, et al. 2023 (92)	Da Vinci SP	Some concerns	High risk	Low risk	Low risk	Low risk	Some concerns	High risk
Oh, et al. 2023 (93)	Da Vinci SP	Some concerns	Low risk	Low risk	Low risk	Low risk	Some concerns	High risk
Tyson, et al. 2023 (94)	Da Vinci SP	Some concerns	High risk	Low risk	Some concerns	Low risk	High risk	High risk

### Overall findings

Demographic data and clinical outcomes of the included studies are presented in Tables 1 and 2.

**Table 1:** Demographics and perioperative outcomes of different procedures performed with novel multiport robotic systems.

First author	Type of study	Type of robotic system	Type of procedure	Number of patients	Age (years), mean (±SD)	Gender, n(%)	Previous abdominal surgery, n(%)	Cohort description	Operative time (min), mean (±SD)	Console time (min), mean (±SD)	Estimated blood loss (ml), mean (±SD)	Intraoperative complications	System malfunction/ instrument conflict	Postoperative complications, n (%)	Length of stay (days), mean (+_SD)	Warm ischemia time (min), mean ± SD	T pathological stang, n(%)	Oncologic outcomes	Functional outcomes
***Hinotori***																			
Miyake, et al. 2023 (24)	Case series Prospective	Hinotori	RAPN transperitoneal	30	62 (median) Range 46-84	20 M (66.6%), 10 F (33.3%)	8 (26.6%)	median tumor size 28 mm, median RENAL score 8	Median 179, range 122-268	Median 106, range 73-191	Median 39, range 5-312	N/A	equipment malfunction requiring recovery time > 5 minutes - 1 case (3.3%)	0>= CD 3	Median 7, range 4-23	Median 13, range 5-20		PSM 0%	median change in eGFR day 1 and 1 month after RAPN were −20.9% and −11.7%
Hinata, et al. 2022 (25)	Case series Retrospective	Hinotori	RARP transperitoneal	30	69.6 (6.24)	30 M (100%)	4 ()13.3%	Mean BMI 23.41 (SD 2.51)	231 (SD 58.12)	167.76 (SD 52.82)	190 (SD 159.9)	N/A	4 recoverable errors, leading to prolonged operative time; recovery time range 8-31 mins	3 (10%) CD>=3 (anastomotic leakage, paralytic ileus, pulmonary embolism)			26 (86.6%) pT2	Overall PSM 4 (13.3%) pT2 PSM 2 (7.7%) pT3 PSM 2 (50%)	
***Hugo-RAS***																			
Totaro, et al. 2023 (26)	Case series Retrospective	Hugo-RAS	RARP transperitoneal	7		7 M (100%)	0%		155 (SD 48.5)	121.9 (SD 37.96)									
Alfano, et al. 2023 (27)	Case series Retrospective	Hugo-RAS	RARP transperitoneal	15	Median 62 (IQR 59-67)	15 M (100%)	0%	Median BMI 24.9 (IQR 23.28), Prostate volume <70g	Median 235 (IQR 213 −271)		Median 300 (IQR 100-310)	0		1 (6.6%)	Median 2 (IQR 2-2)		11 (73.3%) pT2	Overall PSM 5 (33.3%) pT3 PSM 26% undetectable PSA at 4 weeks 100%	1 mo Continence 60%
Balestrazzi, et al. 2024 (28)	Case series Retrospective	Hugo-RAS	RASP transperitoneal	20	Median 72 (IQR 67-76)	20 M (100%		Median BMI 28.5 (IQR 35-30.5)	Median 165 (IQR 121-180)	Median 125 (IQR 101-148)	Median 400 (IQR 300-875)	0	1 (5%) Monopolar curved shear replacement	Overall 3 (15%) CD>=3 - 0	Median 4 (IQR 3-4)				Qmax at first follow-up – median 17.2 (IQR 14-27.6)
Bravi, et al. 2023 (29)	Case series Retrospective	Hugo-RAS	RARP transperitoneal	112	Median 65 (IQR 60-70)	112 M (100%)		Median BMI 26 (IQR 24-29)	Median 180 (IQR 145-200)	Median 150 (IQR 145-175)	Median 400 (IQR 250-575)	0		9 (8%)	Median 3 (IQR 3-4)		78 (69.6%) pT2	Overall PSM 10 (8.9%) Lymph node yield median 15 (IQR 9-19)	1 mo Continence 31.77% 3 mo Continence 71.02%
Elorietta, et al. 2023 (30)	Case series Retrospective	Hugo-RAS	ureteral reimplantation, pyeloplasty, nephrectomy for athrophy, ureterolithotomy	5	50 (SD 13.33)	2 M (40%), 3 F (60%)	3 (60%)	Mean BMI 27.74 (SD 9.16)	211.2 (SD 35.44)	115 (SD 26.61)	19 (SD 6.63)	0		0	3 (SD 1.09)				
Gallioli, et al. 2023 (31)	Case series Prospective	Hugo-RAS	RAPN transperitoneal	10	Median 68, IQR 61-75	6 M (60%), 4 F (40%)		median lesion size 3 cm (IQR 2.2 - 3.7), PADUA score 8 (IQR 8-9)		Median 138, IQR 124-162	Median 90 (IQR 75-100)	0	One conversion to laparoscopy due to hepatomegaly and suboptimal trocar positioning	Overall 3 (30%) CD>=3 – 1 (1%)	Median 4 (IQR 3-6)	Median 13 (IQR 10-14)		PSM 0%	
Mottaran, et al. 2023 (32)	Case series Retrospective	Hugo-RAS	sacropexy	5	Median 73 (IQR 56-76)	5 F (100%)	2 (40%)	Median BMI 25 (IQR 22-28)	Median 130 (IQR 115-165)	Median 80 (IQR 80-115)	Median 20 (IQR 10-35)	0		0	Median 2 (IQR 1-2)				
Raffaeli, et al. 2023 (33)	Case series Retrospective	Hugo-RAS	Adrenalectomy transperitoneal	5	60.6 (SD 16.88)	1 M (20%), 4 F (80%)		Mean BMI 24.66 (SD 3.67) Diagnosis: 3 Cushing, 1 paradrenal tumor, 1 pheochromocytoma; tumor size 30-90 mm	119 (SD 24.94)	61.4 (SD 25.75)		0	Instrument clashes – first procedure	Overall 1 (20%) CD>=3 – 0%	3.2 (SD 2.4)				
Ragavan, et al. 2023 (34)	Case series Retrospective	Hugo-RAS	RARP transperitoneal	17	Median 68 (IQR 66-72)	17 M (100%)	0	Median BMI 24.6 (IQR 22.65-26.63) Pts with bladder neck surgery were excluded	Median 195 (IQR 180-240)	Median 195 (180-240)		0			Median 1 (IQR 1-2)			PSM 0% LN yield median 8.5 (IQR 6.75-10) 1 mo PSA 0.07	1 mo Continence (1 protective pad) 100%
***Kang-DUO***																			
Fan, et al. 2021 (35)	Case series Prospective	Kang-DUO	Pyeloplasty transperitoneal	16	Median 27 (range 21-75)	7 M (43.7%) 9 F (56.2%)		Median BMI 23 (range 15-33) 3 pts had concomitant renal calculi, one horseshoe kidney, one solitary kidney	Median 151 (range 110-190)		Median 8 (range 5-50)			CD>=3 – 0%	Median 4 (range 3-9)				
Fan, et al. 2022 (36)	Case series Prospective	Kang-DUO	RARP extraperitoneal	16	Median 66 (range 58-75)	16 M (100%)	5 (31.2%)	Median BMI 23.62 (range 19.69-28.01) 2 pts had neoadjuvant hormonal therapy; median prostate volume 39g (range 18-86); preop PSA 6.67 ng/ml (range 0.88 - 17.98); all pts cT2		Median 87 (range 70-120)	Median 50 (range 10-200)		No major technical problems	Overall 1 (6.2%) CD>=3 – 0%	Median 5 (range 4-10)		8 (50%) pT2 8 (50%) pT3	Overall PSM 4 (25%)	1 mo continence 14 (87.5%)
Dong, et al. 2023 (37)	Case series Prospective	Kang-DUO	Partial adrenalectomy retroperitoneal	23	Median 49 (range 22-67)	10 M (43.47%), 13 F (56.52%)	6 (26.08%)	Median BMI 23.8 (IQR 20.8-27) 9 pts hormone-active tumor; median diameter of tumor 2.8	Median 86.5 (IQR 60-112.5)	Median 46.5 (IQR 35.1-59)	Median 50 (IQR 20-400)			Overall 3 (13.04%) CD>=3 – 0%	Median 4 (IQR 3-5)			PSM 0%	
Li, et al. 2023 (38)	RCT Prospective	Kang-DUO	RAPN Trans- and retroperitoneal	49	54.36 (SD 10.23)	27 M (55.1%), 22 F (44.89%)	22 (44.89%)	Mean BMI 25.8 (SD 2.84)					no	Overall 3 (6.12%) CD>=3 – 0%		18.38 (SD 5.48)		PSM 0%	
***Revo-I***																			
Alip, et al. 2022 (39)	Case series Prospective	Revo-I	RARP Retzius-sparing	33	71 (SD 6)	33 M (100%)	3 (9.09%)	Mean BMI 24.86 (SD 3.6	126.18 (SD 55.16)	89.45 (SD 31.3)	284.24 (SD 262.33)	0	first 17 pts - survey: physiscians felt most comfortable in porting and docking, found the console and the monitor convenient, were satisfied with total operative time	Overall 3 (9.09%) CD>=3 – 0%	5 (SD 1.87)		27 (81.8%) pT2	PSM 48.48% 6 mo biochemical recurrence 12%	ofr the first 17 cases: continence 58.8% at 1 week, 82.4% at 1 month; at 3 months: 12 patients continent, three pts used one saftey pad, two pts used 2 saftey pads
***Senhance***																			
Kastelan, et al. (2021 40)	Case series Prospective	Senhance	RARP Extraperitoneal	70	Median 65 (IQR 45-70)	70 M (100%)		average prostate volume 40g (20-100)	Median 200 (IQR 120-305)		Median 200 (IQR 100-700)			Overall 6 (8.57%) CD>=3 – 0%	Median 5 (IQR 4-7)		56 (80%) pT2	PSM 18 (25.71%)	
Venckus et al 2021 (41)	Case series Prospective	Senhance	RARP Extraperitoneal	127	Median 61 (IQR 37-73)	127 M (100%)		Median BMI 26.2 (IQR 19-40.1)	Median 180 (IQR 150-215)		Median 250 (IQR 175-430)			Overall 15 (11.81%) CD>=3 – 3 (2.36%) (urethral stricture, lymphocele, vesico-abdominal fistula))			108 (85%) pT2	PSM 43 (33.85%) LN yield median 8 (range 4-24)	
Knezevic et al. 2022 (42)	Case series Retrospective	Senhance	Adrenalectomy transperitoneal	12	Median 48.3 (IQR 42.5 −51.5)	6 M (50%) 6 F (50%)		11 primary hiperaldosteronisn, 1 adrenal cyst (13 cm); mean adenoma size 1.7 cm (IQR 1.3-2)	Median 165.1 (IQR 146.2-188.7)	Median 98.6 (IQR 85-112.5)	Median 47 (IQR 8.75-62.5)	1 conversion to laparoscopy (adhesive perinephric fat)		CD>=3 – 1 (8.33%) (bleeding)	Median 4.5 (IQR 4-5)				
Kulis, et al. 2022 (43)	Case series Prospective	Senhance	RARP Extraperitoneal	107	Median 65 (IQR 60-68)	107 M (100%)			Median 195 (IQR 180-218)		Median 300 (IQR 200-500)	7 conversions due to patients’ anatomy	2 technical issues leading to conversion to laparoscopy	Overall 10 (9.34%) CD>=3 – 1 (0.9%)	Median 5 (IQR 5-5)		87 (81.3%) pT2	PSM 30 (28%) Median LN yield 7 (range 4-16) 9 mo – 3 pts biochmeical recurrence	12 mo continence 79%
Sassani, et al. 2022 (44)	Case series Retrospective	Senhance	Sacropexy	25	62.3 (SD 9.2)	25 F (100%)	13 (52%)	BMI 26.5 (SD 3.8)	210 (SD 48.6)		35 (range 25-50)	0	1 conversion to laparosocpy due to robotic software malfunction	Overall 2 (8%) CD>=3 – 1 (4%)					no anatomical or subjective recurrence at first visit 2pts had anatomical recurrence during follow-up; two pts diagnosed with small bowel obstruction within 30 days of surgery; one pt apical mesh exposure at 4 mnths
***Versius CMR***																			
Hussein, et al. 2022 (45)	Case series Retrospective	Versius CMR	Pyeloplasty	9	Median 30 (IQR 71-34)	6 M (66.6%) 3 F (33.3%)			Median 130 IQR 103-173()		Median 100 (IQR 50-100)	0	0 conversion		Median 3 (IQR 2-3)				
			RARN	10	Median 56 (IQR 47-60)	6 M (60%) 4 F (40%)			Median 167 (IQR 160-190)		Median 200 (100-600)	1 (10%)	1 conversion		Median 3 (IQR 2-4)				
			Siple nephrectomy	42	Median 35 (IQR 25-50)	25 M (59.5%) 17 F (40.4%)			Median 145 (115-170)		Median 100 (50-200)	2 (4.76%)	2 conversions		Median 4				
			Ureterolithotomy	17	Median 39 (24-48)	12 M (70.58%) 5 F (29.4%)			Median 130 (95-160)		Median 28 (5-163))	1 (5.8%)	1 conversion	CD>=3 – 3 (17.6%)	Median 2 (IQR 1-3)				
			RAPN	6	Median 45 (26-50)	3 M (50%) 3 F (50%)			Median 170 (140-180)		Median 450 (150-500)	0	0 conversion						
													Malfunction of the arms in 2 procedures						

BMI = body mass index; RAPN = robotic-assisted partial npehrectomy; RARN = robotic-assisted radical nephrectomy; RARP = robotic-assisted radical prostatectomy; RASP = robotic-assisted simple prostatectomy

**Table 2:** Demographics and perioperative outcomes of different procedures performed with Da Vinci single-port system.

First author	Type of study	Type of procedure	Number of patients	Age (years), mean (±SD)	Gender, n(%)	Previous abdominal surgery, n(%)	Cohort description	Operative time (min), mean (±SD)	Console time (min), mean (±SD)	Estimated blood loss (ml), mean (±SD)	Intraoperative complications	Postoperative complications, n (%)	Length of stay (days), mean (±SD)	Warm ischemia time (min), mean ± SD	T pathological stang, n(%)	Oncologic outcomes	Functional outcomes
Dobbs, et al. 2019 (46)	Case series Prospective	RARP transperitoneal	10	52 (SD 8.3)	10 M (100%)		BMI 29.4 (SD 3.8)	Median 234 (IQR 216-247)	Median 189 (IQR 171-207)	65 (SD 45)	O conversions	0	Median 1 (IQR 1-2)		6 (60%) p T2	PSM 2 (20%)	
Heo, et al. 2019 (47)	Case series Retrospective	Pyeloplasty Transperitoneal	3	47.67 (SD 21.08)	2 M (66.6%) 1 F (33.3%)		BMI 22.8 (SD 4.14)	177.33 (SD 37.03)	149.33 (SD 27.52)		0	0	3.33 (SD 0.47				
Ng, et al. 2019 (48)	Case series Prospective	RARP Transperitoneal	20	67.7 (SD 6)	20 M (100%)			208.9 (SD 35.2)		296.3 (SD 220.7)	0	Overall 5 (25%) CD>=3 - 0	5 (SD 1.7)		11 (55%) pT2	PSM 11 (25%) LN yield 8.3 (SD 7.1)	
Steinberg, et al. 2019 (49)	Case series Retrospective	RARP Transperitoneal	15	Median 62 (range 57-71)	15 M (100%)		BMI 27.6 (SD 3.6)	224 (SD 43)		198 (SD 115)	0	Overall 1 (6.66%) CD>=3 – 1 (6.66%)	Median 1 (IQR 1-2)		10 (66.6%) pT2	PSM 2 (13.3%)	
Steinberg, et al. 2020 (50)	Case series Retrospective	RASP extraperitoneal	10	69 (SD 4)	10 M (100%)			172 (SD 19)		141 (SD 98	0	0	1.1 (SD 0.3)				
Agarwal, et al. 2020 (51)	Case series Retrospective	RARP Transperitoneal and Retzius-spering	49	Median 62 (IQR 58-66)	49 M (100%)	22 (44.89%)	Median BMI 28.5 (25.5-31.7)	Median 161 (IQR 134-194)		Median 200 (IQR 75-300)		Overall 4 (8.16%) CD>= 3 −0	1 (IQR 1-1)		40 (81.63%) pT2	PSM 13 (26.53% LN yield median 8 (IQR 4-11))	3 mo continence 76.19%
Chae Na, et al. 2020 (52)	Case series Retrospective	RAPN Transperitoneal	9	52.88 (SD 9.79)	6 M (66.6%) 3 F (33.3%)	3 (33.3%)		122.66 (SD 16.97)		44.4 (SD 41.12)	1 (11.1%)	0	5.33 (SD 0.81)	27.5 (SD 6.86)		PSM 1 (11.1%)	
Fang, et al. 2020 (53)	Case series Retrospective	RAPN trans and retroperitoneal	13	58.6 ()SD 13.9	12 M (92.3%) 1 F (7.69%)	5 (38.4%)	BMI 31.6 (SD 5.7)	176.1 (SD 64)		Median 200 (IQR 50-800)	1 conversion to open and radical nephrectomy	Overall 3 (23.07%)	1.9 (SD 1.3)				
		RARN trans ansd retroperitoneal	3	61 (SD 1.7)	1 M (33.3%) 2 F (66.6%)	2 (66.6%)	BMI 28.2 (3.3)	176.3 (SD 73.8)		Median 50 (IQR 50-400)	0	Overall 2 (66.6%)	3.3 (SD 1.2)				
Francavilla, et al. 2022 (54)	Case series Retrospective	RARP Transperitoneal	40	Median 61.5 (IQR 58-65)	40 M (100%)		Median BMI 29.6 (IQR 25.4-30.5)	Median 238.5 (IQR 219.3-258)		Median 75 (IQR 50-125)		CD>= 3 – 5 (12.5%)	Median 1 (IQR 1-1)		24 (60%) pT2	PSM 15 (37.5%) Median LN yield 12 (IQR 9-12)	3 mo continence 25 (67.56%)
Ganesan, et al. 2020 (55)	Case series Retrospective	Sacropexy	3	65.67 (SD 1.53)	3 F (100%)		BMI 23.33 (SD 1.53)	225.67 (SD 25.11)		23.3 (SD 18.85)	0	0	1				
Huang, et al. 2021 (56)	Comparative study Retrospective	RARP Transperitoneal	26	Median 63 (IQR 60-66)	26 M (100%)		BMI median 28 (IQR 27-33)	Median 180 (IQR 170-190)				Overall 4 (15.38% CD>= 3 – 1 3.84%)	Median 1 (IQR 1-2)		14 (53.84%) pT2	PSM 6 (23.07%)	
Jones, et al. 2020 (57)	Case series Retrospective	RARP Transperitoneal	23	62 (range 48-77)	23 M (100%)		BMI median 30 (IQR 24.4-47.4)	Median 236 (IQR 191-343)	Median 188.5 (IQR 171-206)		1 (4.34%)	CD>=3 – 6 (26.08%)	Median 1 (IQR 1-2)			PSM 9 (39.13%) Median LN yield 12.5 (range 5-41)	
Kaouk et al , 2020 (58)	Comparative Retrospective	RARP Extraperitoneal	52	62.5 (SD 6)	52 M (100%)		BMI 29.5 (SD 4.9)	201 (SD 37.5)				Overall 6 (11.53%) CD >=3 – 4 (7.69%)	Median 0.17 (IQR 0.13-0.73)		27 (51.9%) pT2	PSM 14 (26.9%)	3 mo continence 59.61%
		RARP Transperitoneal	46	61.1 (SD 6.9)	46 M (100%)		BMI 29.34 (SD 5.3)	248.2 (SD 48.3)				Overall 7 (15.21%) CD>=3 – 3 (6.52%)	Median 1.07 (IQR 1.02-1.82)		27 (58.69% pT2)	PSM 19 (41.3%)	3 mo continence 63%
Kim, et al. 2020 (59)	Case series Retrospective	RARP Transperitoneal	20	Median 66 (IQR 60-71)	20 M (100%)		Median BMI 24 (IQR 22.4-25.7)	Median 245 (IQR 200-255)	Median 190 (IQR 165-210)		0	CD>=3 – 0			11 (55%) pT2	PSM 7 (35%) Median LN yield 19 (IQR 14-22)	
Saidian, et al. 2020 (60)	Comparative Retrospective	RARP Transperitoneal	47	64.7 (SD 7.2)	47 M (100%)		BMI 30.4 (SD 5.4)	255.9 (SD 44.1)		166.2 (SD 114.2)			1.1 (SD 0.5)		20 (42.55%) pT2	PSM 10 (21.27%)	3 mo continence 34 (72.34%)
Shukla, et al. 2021 (61)	Case series Retrospective	RAPN Transperitoneal	12	57.8 (SD 11)	10 M (83.3%) 2 F (16.6%)		BMI 27.9 (SD 4.9)	171.6 (SD 40.5)		68.3 (SD 74.6)	0	CD>=3 - 0	1.2 (SD 0.4)			PSM 1 (8.33%)	
Vigneswaran et al 2020 (62)	Case series Retrospective	RARP Trans and extraperitoneal	50	Median 63 (IQR 58-68)	50 M (100%)		BMI median 30 (IQR 25-32)	Median 290 (IQR 270-320)	Median 230 (IQR 210-250)		0	Overall 7 (14%)	Median 1 (IQR 1-2)		29 (58%) pT2	PSM 21 (42%)	3 mo continence 43 (86%)
Wilson, et al. 2020 (63)	Case series Retrospective	RARP Extraperitoneal	60	63 (SD 6)	60 M (100%)		BMI 30 (SD 4.5)	Median198 (173-222)		179	0	Overall 11 (18.33%) CD>=3 – 7 (11.66%)	0.175		32 (53.55%) pT2	PSM 14 (23.3%)	1 mo continenece 48.27% 3 mo continence 75.67%
Garden, et al. 2021 (64)	Case series Retrospective	Living donor nephrectomy transperitoneal	7	40.3 (SD 15.8)	3 M (42.85%) 4 F (57.14%)		BMI 26.79 (SD 5.57)	218.3 (SD 16.3)		50	0	Overall 2 (28.57%) CD>=3 - 0	2.1 (SD 0.4)				
Lee, et al. 2021 (65)	Matched- comparison Retrospective	Sacropexy	8	66.1 (SD 8)	8 F (100%)	5 (62.5%)	BMI 23.9 (SD 2.5)	141.8 (SD 23.5)	89 (SD 9.53)	71.25 (SD 41.21)	1 (12.5%)	Overall 2 (25%) CD>=3 – 1 (12.5%)					
Lenfant, et al. 2021 (66)	Matched-comparison Retrospective	RARP perineal	26	Median 63 (IQR 59-68)	26 M (100%)	22 (84.61%)	Median BMI 28.3 (IQR 26.4-32.3)	Median 255 (IQR 204-289)		Median 100 (IQR 50-150)		Overall 13 (50%) CD>=3 – 6 (23.07%)	Median 0.95 (IQR 0.83-1.75)		14 (53.84%) pT2	PSM 17 (65.38%) Median LN yield 3 (IQR 1.8-5.2)	3 mo continence 52.63% 12 mo continence 80%
Abou Zeinab, et al. 2022 (67)	Matched-paired comparison Retrospective	RARP Extraperitoneal	78	Median 62.5 (IQR 58.1-67.1))	78 M (100%)		Median BMI 27.4 (IQR 25.5-30.5)	Median 190 (IQR 171-209)				Overall 11 (14.1%) CD>= 3 – 5 (6.41%)	Median 0.19 (IQR 0.15-0.63)			PSM 20 (25.64%)	3 mo continence 80.76%
		RARP Transvesical	78	Median 61.5 (IQR 58.4-66.2)	78 M (100%)		Median BMI 28.3 (IQR 25.5-31.3)	Median 210 (IQR 186-236)				Overall 11 (14.1%) CD>=3 – 2 (2.56%)	Median 0.23 (IQR 0.17-0.91)			PSM 12 (15.38%)	3 mo continence 96.15%
Abou Zeinab, et al. 2022 (68)	Case series Retrospective	RASP Transvesical	91	68.9 (SD 6.7)	91 M (100%)		BMI 28 (SD 4.7)	159.05 (SD 45.57)			2 (2.19%)	10 (10.9%)	0.87 (SD 0.27-1.08)				
Balasubramanian, et al. 2022 (69)	Comparative unmatched Retrospective	RARP Retzius-sparing	32	62 (SD 7.7)	32 M (100%)	4 (12.5%)	BMI 28.7 (SD 3.8)	208 (SD 40)		112 (SD 46)					28 (87.5%) pT2	PSM 10 (31.25%) LN yield 4.5 (SD 2.6)	3 mo continence 93.75%
		RARP Extraperitoneal	30	64.6 (SD 8.6)	30 M (100%)	6 (20%)	BMI 32.1 (SD 6.4)	224 (SD 41)		138 (SD 87)					26 (86.66% pT2)	PSM 8 (26.6%) LN yield 4.5 (SD 4.6)	3 mo continence 51.72%
		RARP Transperitoneal	39	62.7 (SD 6.8)	39 M (100%)	6 (15.38%)	BMI 28.8 (SD 4.3)	248 (SD 36)		130 (SD 70)					21 (53.84%) pT2	PSM 10 (25.64%) LN yield 4.9 (SD 3.4)	3 mo continence 76.92%
Bassett, et al. 2022 (70)	Case series Retrospective	RARP Retzius-sparing	28	65 (SD 7)	28 M (100%)	7 (25%)	BMI 25 (SD 4)	234 (SD 61)				Overall 4 (14.28%) CD>=3 – 2 (7.14%)	0.96 (SD 0.5)		15 (53.57%) pT2	PSM 5 (17.85%)	1 mo continence 82.14% 3 mo continence 89.28%
Beksac, et al. 2022 (71)	Case series Retrospective	Pyeloplasty Transperitoneal	12	42 (SD 24.62)	5 M (41.66%) 7 F (58.33%)		BMI 27.4 (SD 5.64)	159 (SD 30.14)			0	Overall 1 (8.33%) CD>=3 - 0	0.5 (SD 0.4)				
Covas Moschovas, et al. 2022 (72)	Case series Retrospective	RARP Transperitoneal	100	Median 62 (IQR 56-68)	100 M (100%)		Median BMI 25.4 (IQR 23.4-27.4)	Median 114 (IQR 104-124)	Median 80 (IQR 75-90)		0					PSM 15 (15%)	
Fang, et al. 2023 (73)	Comparative unmatched Retrospective	Adrenalectomy Transperitoneal	11	54.2 (SD 8.9)			BMI 31.8 (SD 5.9)	124.6 (SD 38.5)		18 (SD 13)	0	0				PSM 1 (9.09%)	
Francavilla, et al. 2022 (74)	Case series Retrospective	RAPN Transperitoneal	14	Median 54.5 (IQR 48-71)	9 M (64.28%) 4 F (35.71%)		Median BMI 30 (IQR 23.4 −32)	Median 202 (IQR 162-231)		Median 50 (IQR 43-225)	1 (7.14%))	Overall 2 (14.28%) CD>=3 – 2 (14.28%)	Median 1 (IQR 1-2)	Median 18 (IQR 15-24)		PSM 1 (7.14%)	
Ganesan, et al. 2022 (75)	Comparative propensity-matched Retrospective	RASP Transvesical	16	Median 70 (IQR 66-72)	16 M (100%)		Median BMI 28.8 (IQR 24.8-32.1)	Median 176 (IQR 163-195)		Median 100 (IQR 87-150)		1 (6.25%)	Median 1 (IQR 1-1)				
Harrison, et al. 2022 (76)	Comparative propensity-matched Retrospective	Pyeloplasty Transperitoneal	21	41.1 (SD 19.3)			Median BMI 23.7 (21.5-26.8)	Median 128 (IQR 112-145)			1 (4.76%)	Overall 1 (4.76%) CD>=3 - 1 (4.76%)	Median 1 (IQR 1-1)				
Harrison, et al. 2022 (77)	Comparative propensity-matched Retrospective	RARP Extraperitoneal	98	Median 61.9 (IQR 57.4-65.6)	98 M (100%)			Median 147 (IQR 115-193)		50	2 (2.04%)	4 (4.08%)			76 (77.55%) pT2	PSM 17 (17.34%) Mean LN yield 4	12 mo continence 44.89%
Kaouk, et al. 2022 (78)	Case series Retrospective	Partial prostatectomy Transvesical	9	Median 59.4 (IQR 55.1-61.7)	9 M (100%)		Median BMI 28.1 (IQR 27.1-30.9)	Median 208 (Iqr 199-211)	Median 141 (IQR 100-151)	Median 50 (45-100)	0	Overall 2 (22.22%) CD>=3 - 0	Median 0.15 (IQR 0.15-0.16)		5 (55.55%) pT2	PSM 4 (44.44%	
Kaviani, et al. 2022 (79)	Case series Retrospective	RAKT Trans and extraperitoneal	12	55 (SD 10)	6 M (50%) 6 F (50%)		BMI 30 (SD 7)	Median 365 (IQR 318-392)			0	Overall 2 (16.66%) CD>=3 - 0	Median 2 (IQR 2-3)				
Kim, et al. 2022 (80)	Comparative propensity-matched	RAPN Transperitoneal	22	Median 54 (IQR 34.3-61.5))	13 M (59.09%) 9 F (40.9%)		Median BMI 24.4 (IQR 22.7-26.2)	Median 155 (IQR 140-175)				0		Median 26 (IQR 22-32)		PSM 0 %	
Kim, et al. 2022 (81)	Case series Retrospective	RARP Trans and extraperitoneal	157	Median 63 (IQR 59-68)	157 M (100%)		Median BMI 27.8 (IQR 25.8-29.6)	Median 195 (IQR 165-221)		Median 100 (IQR 100-200)	0	Overall 6 (3.82%) CD>=3 – 4 (2.54%)	Range 0-2		119 (75.79%) pT2	PSM 46 (29.29%) Median LN yield 5.9 (IQR 3-7)	12 mo continence 82.8%
Koukouris, et al. 2022 (82)	Case series Prospective	RARP Retzius-sparing	10	Median 70 (IQR 62.5-72)	10 M (100%)		Median BMI 23.5 (IQR 21.3-24.3)	Median 106 (IQR 101-109))	Median 65 (IQR 63-68)	Median 125 (50-150)	0	Overall 1 (10%) CD>=3 - 0	Median 3 (IQR 3-4)		6 (60%) pT2	PSM 5 (50%)	1 mo continence 70% 3 mo continence 100%
Lee, et al. 2022 (83)	Comparative-matched Retrospective	Adrenalectomy Retroperitoneal	8	40.1 (SD 9.8)	4 M (50%) 4F (50%)		BMI 24.8 (SD 3.1)	99 (SD 16.2)	57.1 (SD 15.2)		0	0	2.5 (SD 0.5)				
Levy, et al. 2023 (84)	Case series Retrospective	RARP Extraperitoneal and transvesical	14	Median 64 (IQR 61-66)	14 M (100%)	13 (92.85%)	Median BMI 27 (IQR 25-29)	Median 282 (IQR 254-308)		Median 100 (IQR 56-150)	0	Overall 1 (7.14%) CD>=3 – 1 (7.14%)	Median 1 (IQR 1-1)		8 (57.14%) pT2	PSM 4 (28.57%)	1 mo continence 50%
Liu, et al. 2022 (85)	Case series Retrospective	Posterior urethroplasty Transvesical	9	65.4	9 M (100%)	8 (88.88%)	BMI mean 30.5	377 (range 257-534)			0	Overall 5 (55.55%) CD>=3 – 3 (33.33%)	Median 2 (range 0-4)				
Noh, et al. 2022 (86)	Case series Retrospective	RARP Transperitoneal	31	68.5 (SD 6.3)	31 M (100%)		BMI 24.6 (SD 2.8)	151.3 (SD 15.1)	111 (SD 15.7)	121.1 (SD 140.5)		0			22 (70.96%) pT2	PSM 6 (19.35%)	3 mo continence 77.41%
Palacios, et al. 2022 (87)	Case series Retrospective	RAPN Retroperitoneal	20	Median 56.5 (IQR 48.5-65))			Median BMI 30.75 (IQR 25.2-38.8)	Median 166.5 (IQR 135.5-201)				Overall 2 (10%) CD>=3 – 1 (5%)	2.7 (SD 3.4)	Median 25 (IQR 17-27)			
Ramos-Carpinteyro, et al. 2022 (88)	Case series Retrospective	RARP Transvesical	100	Median 62.1 (IQR 58.1-66.3)	100 M (100%)	49 (49%)	Median BMI 28.1 (IQR 25.2-31.3)	Median 212.5 (IQR 188-238.8)		Median 100 (IQR 50-100)	0	Overall 16 (16%) CD>=3 – 2 (2%)	Median 0.23 (IQR 0.04-0.91)		62 (62%) pT2	PSM 15 (15%)	3 mo continence 87%
Bang, et al. 2023 (89)	Comparative unmatched Retrospective	RAPN Trans- and retroperitoneal	30	50.13 (SD 11.97)			BMI 24.75 (SD 2.88)	108.07 (SD 43.19)	67.97 (SD 24.06)			Overall 1 (3.33%)		11.59 (SD 7.31)		PSM 0%	
Harrison, et al. 2023 (90)	Comparative – propensity matched Retrospective	RAPN Trans- and retroperitoneal	40	Median 59.5 (IQR 51.5-70)				Median 102 (IQR 79-127.5)			0	Overall 1 (2.08%%)	Median 1 (IQR 1-1)			PSM 3 (6.25%)	
Kaouk, et al. 2023 (91)	Case series Retrospective	RAKAT transperitoneal	8					Range: 366-701				0	Median 3				
Kim, et al. 2023 (92)	Case series Retrospective	RARP Transperitoneal	66	Median 68 (range 48-78)	66 M (100%)		Median BMI 24.2 (Range 17.2-30.1)	Median 210 (Range 130-390)	Median 130 (Range 90-340)	Median 200 (Range 50-1000)	0	Overall 1 (1.51%) CD>=3 – 1 (1.51%)	Median 7 (Range 3-9)		38 (57.57%) pT2	PSM 23 (34.84%) Median LN yield 19 (range 4-34)	
Oh, et al. 2023 (93)	Case series Retrospective	Sacropexy	66	63.7 (SD 7.6)	66 F (100%)		BMI 24.9 (SD 3.2)	201.5			4 (6.06%)		Median 4 (IQR 2-7)				
Tyson, et al. 2023 (94)	Case series Retrospective	RARC	41	Median 70.4 (IQR 64.2-73.8)			Median BMI 25.2 (IQR 23.5-27.8)	Median 480 (IQR 432-528)			17 conversions	11/24 (45.83%)	Median 7(IQR 5-8.2)			PSM 1/24 (4.16%)	

RAKT = robotic-assisted kidney transplantation; RAKAT = robotic-assisted kidney autotransplantation; RAPN = robotic-assisted partial nephrectomy; RARC = robotic-assisted radical cystectomy; RARP = robotic-assisted radical prostatectomy; RASP= robotic-assisted simple prostatectomy

The most frequent procedures performed with new robotic systems were robotic-assisted radical prostatectomy (RARP) (Hinotori, Hugo-RAS, Revo-I, Senhance), followed by robotic-assisted partial nephrectomy (RAPN) (Hinotori, Kang-DUO, Versius).

Da Vinci SP system has been extensively used for all urologic procedures in the US, from RARP to robotic-assisted kidney autotransplantation (RAKAT). For RARP, several new types of approach have been developed, such as transvesical and perineal.

### Radical Prostatectomy

**Figure 2:**
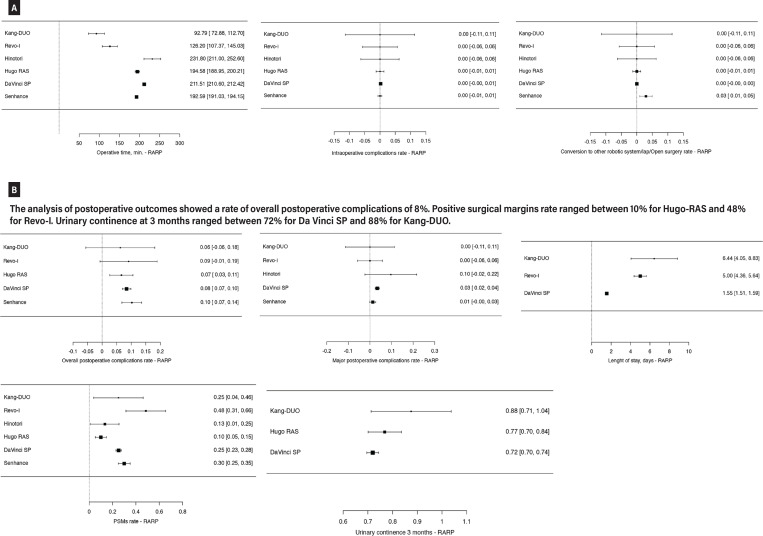
Metaanalysis of perioperative outcomes of RARP performed with new robotic systems. a) The analysis of intraoperative outcomes shows that mean operative time was around 200 mins. The rate of intraoperative complications and conversion to other system or open surgery was low for all systems.

Focusing on evidence reporting perioperative results on RARP, a total of 1879 patients were analyzed (all data are depicted in Figures 2a and b). In particular, 1345 RARP were performed with Da Vinci SP (46, 48, 49, 51, 54, 56-60, 62, 63, 66, 67, 69, 70, 72, 77, 81, 82, 84, 86, 88, 92), 304 with Senhance system (40, 41, 43), 151 with Hugo RAS (26, 27, 29, 34), 33 with Revo-I (39), 30 with Hinotori (25) and 16 with Kang-DUO (36). The operative time was around 200 minutes for the majority of robotic platforms, ranging from a mean of 96.79 minutes for Kang-DUO to a maximum of 231.8 minutes for Hinotori. Only 3 intraoperative complications were recorded (all in the Da Vinci SP group, 0.22%) and only 9 conversions were recorded (all in the Senhance group, 2.9%). Overall postoperative complications rate was around 8%, whilst major postoperative complications rate ranged from 1%, for Revo-I and Senhance groups to 10% in case of Hinotori series. Overall, the length of stay was underreported; it ranged from a minimum of 1.55 days in the Da Vinci series to 5 and 6.4 days for Revo-I and Kang-Duo groups, respectively.

Focusing on oncological outcome, the PSM rate was 10% in case of Hugo RAS, 13% in case of Hinotori, 25% in case of Da Vinci SP and Kang-DUO, 30% in case of Senhance and 48% in case of Revo-I. Finally, the functional outcomes were underreported. The urinary continence at 3 months of follow-up was 72% in Da vinci SP series, 77% in Hugo-RAS group and 88% in Kang-DUO group. Post-operative erectile function was not reported in these preliminary series.

### Partial Nephrectomy

**Figure 3:**
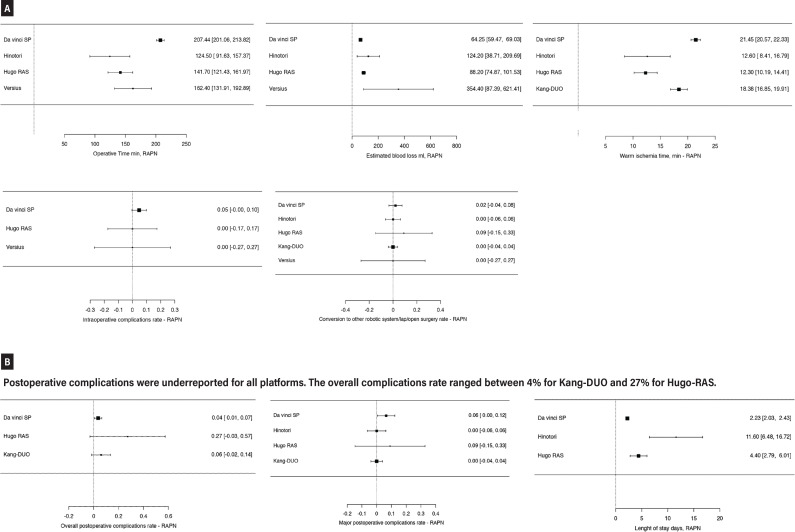
Meta-analysis of perioperative outcomes of RAPN performed with new robotic systems. a) The operative time varied between 124.5 mins for Hinotori and 207.4 mins for DaVinci SP, with WIT between 12 mins for Hinotori and Hugo-RAS groups and 21.45 mins for Kang-DUO and DaVinci SP groups. The EBL ranged between 64.25 ml for DaVinci SP and 354.40 ml for Versius.

A total of 255 patients who underwent RAPN were analyzed, with all data depicted in Figures 3a and b. Specifically, 160 RAPN procedures were performed with Da Vinci SP (52, 53, 61, 74, 80, 87, 89, 90), 49 with Kang-DUO (38), 30 with Hinotori (24), 10 with Hugo-RAS (31), and 6 with Versius (45). The operative time varied, with a mean of 124.5 minutes for Hinotori and up to 207.4 minutes for Da Vinci SP.

Mean EBL was 64.25 mL for the Da Vinci SP series and 88.20 mL for the Hugo-RAS group, reaching a maximum of 354.40 mL in the Versius group. The mean warm ischemia time was approximately 12 minutes for both the Hinotori and Hugo-RAS series and increased to 18.38 minutes and 21.45 minutes for the Kang-DUO and Da Vinci SP groups, respectively.

Intraoperative complications were generally underreported; the Da Vinci SP series reported a 5% rate of intraoperative complications, while no events were recorded in the other series that reported this data (Hugo-RAS and Versius). Postoperative complications were also underreported by all robotic platforms. The overall postoperative complication rate was 4% for Kang-DUO and 6% for Da Vinci SP, increasing to 27% in the Hugo-RAS group. Major postoperative complications ranged from 6% in the Da Vinci SP series to 10% in the Hugo-RAS series, with no events reported in the Hinotori and Kang-DUO series.

Finally, the length of stay ranged from a minimum of 2.23 days in the Da Vinci SP series to 4 days in the Hugo-RAS group and 11.6 days for the Hinotori series.

### Simple prostatectomy

**Figure 4:**
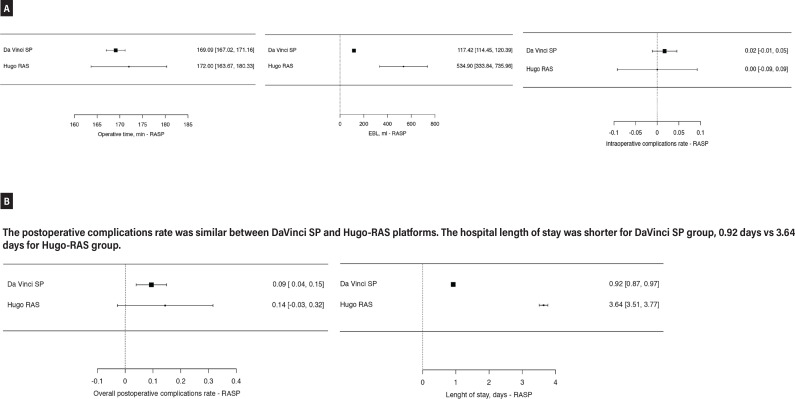
Meta-analysis of perioperative outcomes of RASP performed with new robotic systems. a) DaVinci SP system had a shorter operative time, lower EBL and slightly higher intraoperative complications rate as compared to Hugo-RAS system.

A total of 127 patients who underwent Robot-Assisted Simple Prostatectomy (RASP) were included in the analysis (Figures 4a and b). The majority of procedures (n=107, 100%) were performed using the Da Vinci SP system by transvesical approach (68, 75), with an average operative time of 169.09 minutes. The mean estimated blood loss (EBL) was 117.42 mL. Two (2.2%) intraoperative complications occured among 91 patients with available data. The overall postoperative complication rate was 10.2% (11/107 patients), with an average hospital stay of 0.92 days. The Hugo RAS system was used in 20 cases (28), with a slightly longer operative time of 172 minutes and a significantly higher mean EBL of 534.9 mL. No intraoperative complications were reported in the Hugo group, but the overall postoperative complication rate was 15% (3 patients), and the average length of stay was 3.64 days.

### Other surgeries

**Supplementary Figure 5:**
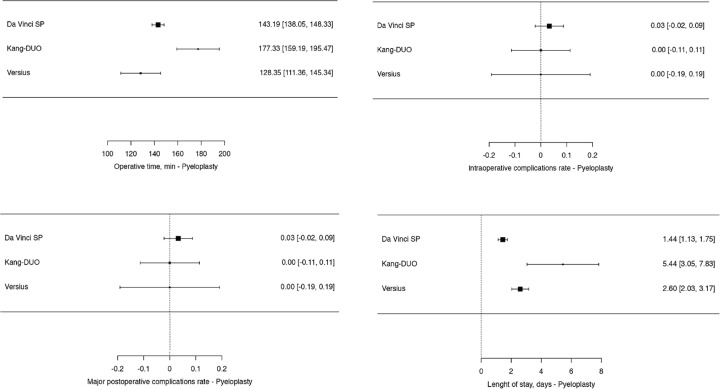
Meta-analysis of perioperative outcomes of pyeloplasty performed with new robotic systems.

**Supplementary Figure 6:**
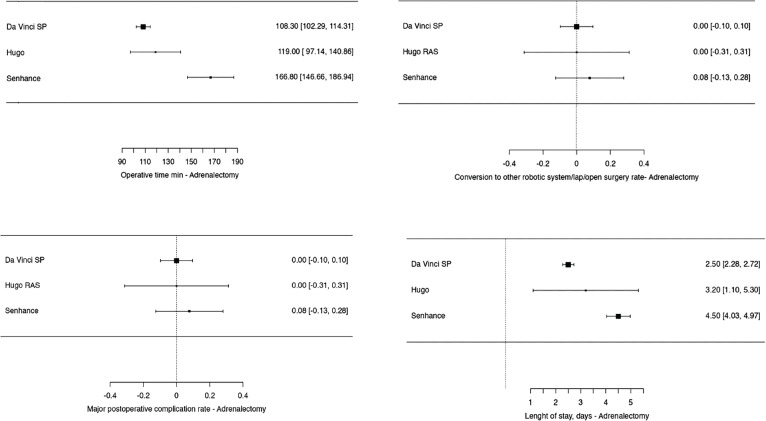
Meta-analysis of perioperative outcomes of adrenalectomy performed with new robotic systems.

**Supplementary Figure 7:**
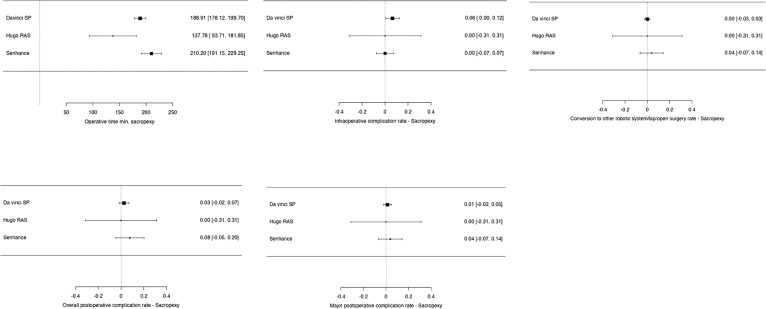
Meta-analysis of perioperative outcomes of sacropexy performed with new robotic systems.

For the analysis of perioperative outcomes related to other urologic procedures, including pyeloplasty, adrenalectomy, and sacropexy, a total of 228 patients were evaluated (Supplementary Figures 5, 6 and 7). The findings are summarized as follows:

**Pyeloplasty:** A total of 61 patients underwent robotic-assisted pyeloplasty. The majority of these procedures (36) were performed using the Da Vinci SP system (47, 71, 76), with an average operative time of 143.19 minutes. Intraoperative complications occurred in one patient (2.77%), and major postoperative complications were also reported in one patient (2.77%). The average length of hospital stay was 1.44 days. The Kang-DUO system was used in 16 cases (35), with an operative time of 177.33 minutes, and no complications were reported. The length of stay was significantly longer, averaging 5.44 days. The Versius platform was used in 9 cases (45), with an operative time of 128.35 minutes and no reported complications, with an average length of stay of 2.6 days.
**Adrenalectomy:** In the case of adrenalectomy, 36 procedures were analyzed. The Da Vinci SP system was used in 19 cases (73, 83), with an average operative time of 108.3 minutes. There were no reported conversions or major complications, and the average length of stay was 2.5 days. The Hugo RAS system was employed in 5 cases (33), with an operative time of 119 minutes, also with no reported complications or conversions, and a mean length of stay of 3.2 days. The Senhance system was used in 12 cases (42), with an operative time of 166.8 minutes, 1 conversion to another surgical approach, and 1 major postoperative complication. The average hospital stay for patients in the Senhance group was 4.5 days.
**Sacropexy:** A total of 107 sacropexy procedures were reviewed. The Da Vinci SP system accounted for 77 cases (55,65,93), with an operative time of 188.91 minutes. Intraoperative complications occurred in 5 patients (6.5%), with 2 postoperative complications (2.6%), and 1 major complication (1.3%). There were no conversions to other surgical approaches. The Hugo system was used in 5 cases (32), with a shorter operative time of 137.78 minutes and no reported complications or conversions. The Senhance system was used in 25 cases (44), with an operative time of 210.2 minutes, 1 conversion, 2 postoperative complications (8%), and 1 major complication (4%).

## DISCUSSION

After Intuitive™'s patents drop in 2019, we have seen several new robotic platforms and technologies in the market, in this context. This systematic review evaluated the early clinical outcomes and safety profile of novel multi-port, single-port and modular robotic systems in urologic surgery. The results reveal promising but varied outcomes across different platforms, highlighting both the potential benefits and challenges of these technologies.

The increasing adoption of new robotic systems is driven by several factors, including the desire to enhance surgical precision, reduce invasiveness, and improve patient outcomes. Novel multi-port systems such as Hugo-RAS, Versius, and others have introduced innovations like open consoles for better communication, modular designs for flexible arm positioning, and enhanced ergonomics. Single-port systems, exemplified by the Da Vinci SP, offer the potential for even less invasive procedures and innovative surgical approaches, such as transvesical radical prostatectomy. These advancements reflect a broader trend in urologic surgery towards more sophisticated and patient-centered technologies.

Radical prostatectomy (RARP) outcomes varied across different platforms. Operative times ranged broadly, with platforms like Hinotori (25) exhibiting longer durations compared to others like Kang-DUO (36), which reported significantly shorter times. Despite these variations, the majority of systems demonstrated acceptable rates of intraoperative and postoperative complications. Notably, the Da Vinci SP system (46, 48, 49,51, 54, 56-60, 62, 63, 66, 67, 69, 70, 72, 77, 81, 82, 84, 86, 88, 92), Kang-DUO (36), Senhance (40, 41, 43) and Revo-I (39) platforms showed a higher rate of positive surgical margins. This data could be related to a wider spread in case of single-port platform (released in 2017), or due to including less selected patients even with advanced/high risk tumors.

Partial nephrectomy outcomes also highlighted the variability in operative times and EBL across different systems. Considering the intra- and postoperative complications rates, they were generally underreported making difficult to proceed with any comparison. Moreover, the relatively low sample size of some platforms, especially in the more recent ones, increase this issue. Despite this limit, novel systems showed promising results with generally acceptable perioperative outcomes.

The progressively wider adoption of these new robotic platforms will underline pros and cons of each system, enhancing the importance of system-specific considerations in clinical practice. For instance, the Hugo-RAS system, despite being a versatile and widely adopted platform (95, 96), reported higher EBL and postoperative complication rates in certain procedures like simple prostatectomy (28). Similarly, the Revo-I system, while effective in reducing operative times in RARP, reported a notably high PSM rate (39), which could be attributed to the learning curve associated with its use or intrinsic system limitations.

In contrast, platforms like Kang-DUO and Hinotori, though less prevalent, demonstrated strong performance in specific procedures like RAPN (24, 38) and RARP (25, 36), with low complication rates and favorable operative times. These findings suggest that while the more established systems like Da Vinci SP are widely used, emerging platforms may offer comparable, if not superior, outcomes in certain contexts.

While the initial results of these novel robotic platforms are encouraging (97, 98), the review highlights several challenges that must be addressed to optimize their integration into clinical practice. The variability in outcomes across different platforms suggests a need for further studies to establish standardized protocols of adoption and training programs that can mitigate learning curves and enhance the consistency of surgical outcomes.

Additionally, the underreporting of functional outcomes, such as urinary continence post-RARP, highlights a gap in the current literature that future studies should address. Understanding the long-term functional outcomes associated with these novel platforms is crucial for fully assessing their impact on patient quality of life.

Moreover, the major drawback of robotic surgery is represented by the costs of the system, instruments and disposable materials. One of the aims of these new platforms was to decrease these costs in order to enable a wider adoption. Several cost analyses have been performed showing that these novel robotic systems can decrease the costs per procedure with 707$ in case of Da Vinci SP (99), 251.31€ for Hugo-RAS (100) and 908.33$ for Senhance (101) as compared to Da Vinci Xi system. However, the cost reduction needs to be balanced with regards to patient outcomes and other drivers of cost, such as facility background and caseload. As such, further studies are necessary to assess the actual economic impact of these new robotic platforms.

Our study has certain limitations that should be acknowledged. First, the inclusion of results and experiences from multiple centers and surgeons makes it challenging to discern whether the observed outcomes were influenced by the robotic platform itself or by varying levels of surgeon expertise. Additionally, when evaluating the costs associated with each platform, it is important to consider that hospital contracts and pricing structures can differ significantly across countries and institutions. Moreover, the literature review primarily consists of retrospective studies, which are subject to inherent risks of bias. There remains a need for well-designed, prospective studies that can provide more robust and comparative data on these robotic platforms. The current paper brings a preliminary review of clinical outcomes of procedures performed with new robotic platforms. However, several other robotic platforms and telesurgery have become available in this rapidly growing market and further reports are awaited. Despite these limitations, this study offers a comprehensive and in-depth analysis of the latest robotic technologies introduced globally, contributing valuable insights to this rapidly evolving field.

## CONCLUSION

The adoption of novel multi-port, single-port and modular robotic systems in urologic surgery is transforming the landscape of surgical care, offering new opportunities for enhanced precision, reduced invasiveness, and potentially improved patient outcomes. All these new systems showed acceptable peri-operative outcomes and good safety profile. However, the variability in perioperative and oncologic outcomes across different platforms underscores the need for continued research, standardized training, and system refinement. As these technologies evolve, they hold the promise of setting new standards in minimally invasive urologic surgery, ultimately benefiting both surgeons and patients.

## Appendix

### FILE 1

#### PubMed

("Robotic Surgical Procedures"[Mesh] OR "Robotics"[Mesh] OR robot*[tiab]) AND (Hugo*[tiab] OR Versius*[tiab] OR Avatera*[tiab] OR Revo-I*[tiab] OR Senhance*[tiab] OR Hinotori*[tiab] OR Dexter*[tiab] OR KD-SR-01*[tiab] OR KangDuo-Surgical-Robot-01*[tiab] OR single-port[tiab] OR (Da-Vinci*[tiab] OR Davinci*[tiab] AND SP[tiab]) OR ((new[title] OR novel[title] OR innovative[title]) AND (platform*[title] OR system*[title]))) AND ("Urogenital System"[Mesh] OR "Adrenal Glands"[Mesh] OR "Urogenital Diseases"[Mesh] OR "Pelvic Organ Prolapse"[Mesh] OR "Adrenal Gland Diseases"[Mesh] OR "Urogenital Surgical Procedures"[Mesh] OR "Adrenalectomy"[Mesh] OR urogen*[tiab] OR uro-gen*[tiab] OR genitourinar*[tiab] OR urol*[tiab] OR prostat*[tiab] OR urinar*[tiab] OR urethr*[tiab] OR transurethr*[tiab] OR suburethr*[tiab] OR ureter*[tiab] OR nephroureter*[tiab] OR urothel*[tiab] OR bladder[tiab] OR colposuspen*[tiab] OR cystectom*[tiab] OR cystoscop*[tiab] OR cystotom*[tiab] OR cystostom*[tiab] OR "ileal conduit"[tiab] OR kidney*[tiab] OR renal[tiab] OR nephro*[tiab] OR nephrectom*[tiab] OR pyelo*[tiab] OR pelvi*[tiab] OR cystocele*[tiab] OR sacropex*[tiab] OR sacrocolpopex*[tiab] OR colposacropex*[tiab] OR "sacral colpopex*"[tiab] OR adrenal*[tiab] OR retroperitoneal*[tiab] OR retroperitoneal*[tiab]) AND ("2016"[Date - Publication] : "3000"[Date - Publication])

**497 risultati al 15/5/2023 (33/33 articoli GS^1^ catturati)**

**Table:**

#1	"Robotic Surgical Procedures"[Mesh] OR "Robotics"[Mesh] OR robot*[tiab]
#2	Hugo*[tiab] OR Versius*[tiab] OR Avatera*[tiab] OR Revo-I*[tiab] OR Senhance*[tiab] OR Hinotori*[tiab] OR Dexter*[tiab] OR KD-SR-01*[tiab] OR KangDuo-Surgical-Robot-01*[tiab] OR single-port[tiab]
#3	Da-Vinci*[tiab] OR Davinci*[tiab] AND SP[tiab]
#4	(new[title] OR novel[title] OR innovative[title]) AND (platform*[title] OR system*[title])
#5	#2 OR #3 OR #4
#6	#1 AND #5
#7 "Urogenital System"[Mesh] OR "Adrenal Glands"[Mesh] OR "Urogenital Diseases"[Mesh] OR "Pelvic Organ Prolapse"[Mesh] OR "Adrenal Gland Diseases"[Mesh] OR "Urogenital Surgical Procedures"[Mesh] OR "Adrenalectomy"[Mesh]
#8	urogen*[tiab] OR uro-gen*[tiab] OR genitourinar*[tiab] OR urol*[tiab] OR prostat*[tiab] OR urinar*[tiab] OR urethr*[tiab] OR transurethr*[tiab] OR suburethr*[tiab] OR ureter*[tiab] OR nephroureter*[tiab] OR urothel*[tiab] OR bladder[tiab] OR colposuspen*[tiab] OR cystectom*[tiab] OR cystoscop*[tiab] OR cystotom*[tiab] OR cystostom*[tiab] OR "ileal conduit"[tiab] OR kidney*[tiab] OR renal[tiab] OR nephro*[tiab] OR nephrectom*[tiab] OR pyelo*[tiab] OR pelvi*[tiab] OR cystocele*[tiab] OR sacropex*[tiab] OR sacrocolpopex*[tiab] OR ccolposacropex*[tiab] OR "sacral colpopex*"[tiab] OR adrenal*[tiab] OR retroperitoneal*[tiab] OR retro-peritoneal*[tiab]
#9	#7 OR #8
#10	#6 AND #9
#11	#10 AND ("2016"[Date - Publication] : "3000"[Date - Publication])

#### Embase

(‘robot assisted surgery’/exp OR ‘robotics’/exp OR ‘robotic surgical device’/exp OR robot*:ti,ab,kw) AND (Hugo*:ti,ab,kw OR Versius*:ti,ab,kw OR Avatera*:ti,ab,kw OR Revo-I*:ti,ab,kw OR Senhance*:ti,ab,kw OR Hinotori*:ti,ab,kw OR Dexter*:ti,ab,kw OR KD-SR-01*:ti,ab,kw OR KangDuo-Surgical Robot-01*:ti,ab,kw OR single-port:ti,ab,kw OR (Da-Vinci*:ti,ab,kw OR Davinci*:ti,ab,kw AND SP:ti,ab,kw) OR ((new:ti OR novel:ti OR innovative:ti) AND (platform*:ti OR system*:ti))) AND (‘urogenital system’/exp OR ‘adrenal gland’/exp OR ‘urogenital tract disease’/exp OR ‘pelvic organ prolapse’/exp OR ‘adrenal disease’/exp OR ‘urologic surgery’/exp OR ‘colposacropexy’/exp OR ‘adrenal surgery’/exp OR urogen*:ti,ab,kw OR uro-gen*:ti,ab,kw OR genitourinar*:ti,ab,kw OR urol*:ti,ab,kw OR prostat*:ti,ab,kw OR urinar*:ti,ab,kw OR urethr*:ti,ab,kw OR transurethr*:ti,ab,kw OR suburethr*:ti,ab,kw OR ureter*:ti,ab,kw OR nephroureter*:ti,ab,kw OR urothel*:ti,ab,kw OR bladder:ti,ab,kw OR colposuspen*:ti,ab,kw OR cystectom*:ti,ab,kw OR cystoscop*:ti,ab,kw OR cystotom*:ti,ab,kw OR cystostom*:ti,ab,kw OR ‘ileal conduit’:ti,ab,kw OR kidney*:ti,ab,kw OR renal:ti,ab,kw OR nephro*:ti,ab,kw OR nephrectom*:ti,ab,kw OR pyelo*:ti,ab,kw OR pelvi*:ti,ab,kw OR cystocele*:ti,ab,kw OR sacropex*:ti,ab,kw OR sacrocolpopex*:ti,ab,kw OR colposacropex*:ti,ab,kw OR ‘sacral colpopex*’:ti,ab,kw OR adrenal*:ti,ab,kw OR retroperitoneal*:ti,ab,kw OR retro-peritoneal*:ti,ab,kw) AND [2016-2023]/py

**756 risultati al 15/5/2023**

**Table:**

#1	‘robot assisted surgery’/exp OR ‘robotics’/exp OR ‘robotic surgical device’/exp OR robot*:ti,ab,kw
#2	Hugo*:ti,ab,kw OR Versius*:ti,ab,kw OR Avatera*:ti,ab,kw OR Revo-I*:ti,ab,kw OR Senhance*:ti,ab,kw OR Hinotori*:ti,ab,kw OR Dexter*:ti,ab,kw OR KD-SR-01*:ti,ab,kw OR KangDuo-Surgical-Robot-01*:ti,ab,kw OR single-port:ti,ab,kw
#3	Da-Vinci*:ti,ab,kw OR Davinci*:ti,ab,kw AND SP:ti,ab,kw
#4	(new:ti OR novel:ti OR innovative:ti) AND (platform*:ti OR system*:ti)
#5	#2 OR #3 OR #4
#6	#1 AND #5
#7	‘urogenital system’/exp OR ‘adrenal gland’/exp OR ‘urogenital tract disease’/exp OR ‘pelvic organ prolapse’/exp OR ‘adrenal disease’/exp OR ‘urologic surgery’/exp OR ‘colposacropexy’/exp OR ‘adrenal surgery’/exp
#8	urogen*:ti,ab,kw OR uro-gen*:ti,ab,kw OR genitourinar*:ti,ab,kw OR urol*:ti,ab,kw OR prostat*:ti,ab,kw OR urinar*:ti,ab,kw OR urethr*:ti,ab,kw OR transurethr*:ti,ab,kw OR suburethr*:ti,ab,kw OR ureter*:ti,ab,kw OR nephroureter*:ti,ab,kw OR urothel*:ti,ab,kw OR bladder:ti,ab,kw OR colposuspen*:ti,ab,kw OR cystectom*:ti,ab,kw OR cystoscop*:ti,ab,kw OR cystotom*:ti,ab,kw OR cystostom*:ti,ab,kw OR ‘ileal conduit’:ti,ab,kw OR kidney*:ti,ab,kw OR renal:ti,ab,kw OR nephro*:ti,ab,kw OR nephrectom*:ti,ab,kw OR pyelo*:ti,ab,kw OR pelvi*:ti,ab,kw OR cystocele*:ti,ab,kw OR sacropex*:ti,ab,kw OR sacrocolpopex*:ti,ab,kw OR colposacropex*:ti,ab,kw OR ‘sacral colpopex*’:ti,ab,kw OR adrenal*:ti,ab,kw OR retroperitoneal*:ti,ab,kw OR retro-peritoneal*:ti,ab,kw
#9	#7 OR #8
#10	#6 AND #9
#11	#10 AND [2016-2023]/py

#### Scopus

**Table:**

#1	TITLE-ABS-KEY(robot*)
#2	TITLE-ABS-KEY(Hugo* OR Versius* OR Avatera* OR Revo-I* OR Senhance* OR Hinotori* OR Dexter* OR KD-SR-01* OR KangDuo-Surgical-Robot-01* OR single-port)
#3	TITLE-ABS-KEY(Da-Vinci* OR Davinci* AND SP)
#4	TITLE(new OR novel OR innovative) AND TITLE(platform* OR system*)
#5	#2 OR #3 OR #4
#6	#1 AND #5
#7	TITLE-ABS-KEY(urogen* OR uro-gen* OR genitourinar* OR urol* OR prostat* OR urinar* OR urethr* OR transurethr* OR suburethr* OR ureter* OR nephroureter* OR urothel* OR bladder OR colposuspen* OR cystectom* OR cystoscop* OR cystotom* OR cystostom* OR "ileal conduit" OR kidney* OR renal OR nephro* OR nephrectom* OR pyelo* OR pelvi* OR cystocele* OR sacropex* OR sacrocolpopex* OR colposacropex* OR "sacral colpopex*" OR adrenal* OR retroperitoneal* OR retro-peritoneal*)
#8	#6 AND #7
#9	#8 AND (PUBYEAR > 2015)

**601 risultati al 15/5/2023**

#### Web of Science

**Table:**

#1	TS=robot*
#2	TS=(Hugo* OR Versius* OR Avatera* OR Revo-I* OR Senhance* OR Hinotori* OR Dexter* OR KD-SR-01* OR KangDuo-Surgical-Robot-01* OR single-port)
#3	TS=(Da-Vinci* OR Davinci* AND SP)
#4	TI=(new OR novel OR innovative) AND TI=(platform* OR system*)
#5	#2 OR #3 OR #4
#6	#1 AND #5
#7	TS=(urogen* OR uro-gen* OR genitourinar* OR urol* OR prostat* OR urinar* OR urethr* OR transurethr* OR suburethr* OR ureter* OR nephroureter* OR urothel* OR bladder OR colposuspen* OR cystectom* OR cystoscop* OR cystotom* OR cystostom* OR "ileal conduit" OR kidney* OR renal OR nephro* OR nephrectom* OR pyelo* OR pelvi* OR cystocele* OR sacropex* OR sacrocolpopex* OR colposacropex* OR "sacral colpopex*" OR adrenal* OR retroperitoneal* OR retro-peritoneal*)
#8	#6 AND #7
#9	Limit #8 to publication years 2016-2023

**1071 risultati al 15/5/2023**

**Totale library deduplicata = 1634 risultati**
